# Loss of DNA Mismatch Repair Imparts a Selective Advantage in Planarian Adult Stem Cells

**DOI:** 10.1371/journal.pone.0021808

**Published:** 2011-07-01

**Authors:** Jessica P. Hollenbach, Alissa M. Resch, Dasaradhi Palakodeti, Brenton R. Graveley, Christopher D. Heinen

**Affiliations:** 1 Neag Comprehensive Cancer Center and Center for Molecular Medicine, University of Connecticut Health Center, Farmington, Connecticut, United States of America; 2 Department of Genetics and Developmental Biology, University of Connecticut Stem Cell Institute, University of Connecticut Health Center, Farmington, Connecticut, United States of America; University of Massachusetts Medical School, United States of America

## Abstract

Lynch syndrome (LS) leads to an increased risk of early-onset colorectal and other types of cancer and is caused by germline mutations in DNA mismatch repair (MMR) genes. Loss of MMR function results in a mutator phenotype that likely underlies its role in tumorigenesis. However, loss of MMR also results in the elimination of a DNA damage-induced checkpoint/apoptosis activation barrier that may allow damaged cells to grow unchecked. A fundamental question is whether loss of MMR provides pre-cancerous stem cells an immediate selective advantage in addition to establishing a mutator phenotype. To test this hypothesis in an *in vivo* system, we utilized the planarian *Schmidtea mediterranea* which contains a significant population of identifiable adult stem cells. We identified a planarian homolog of human *MSH2*, a MMR gene which is mutated in 38% of LS cases. The planarian *Smed-msh2* is expressed in stem cells and some progeny. We depleted *Smed-msh2* mRNA levels by RNA-interference and found a striking survival advantage in these animals treated with a cytotoxic DNA alkylating agent compared to control animals. We demonstrated that this tolerance to DNA damage is due to the survival of mitotically active, MMR-deficient stem cells. Our results suggest that loss of MMR provides an *in vivo* survival advantage to the stem cell population in the presence of DNA damage that may have implications for tumorigenesis.

## Introduction

Lynch syndrome (LS), also referred to as hereditary non-polyposis colorectal cancer, is a hereditary disease that leads to increased risk of early-onset colorectal and other cancers and is caused by germline mutations in DNA mismatch repair (MMR) genes [1]. LS is an autosomal dominant disorder in which the patient inherits one mutant allele and loses the remaining wild-type copy in the tumor. A fundamental question is what happens to the cell upon loss of the remaining wild-type allele such that cancer develops? One hypothesis is that the resultant loss of MMR function leads to a cell with a mutator phenotype, accelerating the tumorigenic process [2]. It is not clear, however, whether this cell, upon loss of MMR function, acquires a selective advantage prior to developing further mutations in growth control and survival genes. In addition to repair, the MMR proteins activate cell cycle checkpoints and apoptosis in response to DNA damage in tissue culture experiments [3]. Thus, we hypothesize that the MMR-defective cell in vivo will have a growth and/or survival advantage over neighboring cells under conditions that give rise to MMR-sensitive damage.

We are interested in the effects of MMR loss in the adult stem cell *in vivo*. Focus on the adult stem cell as the tumor cell of origin has increased in the past decade [4], [5]. To address the effects of MMR loss in adult stem cells, we examined the planarian *Schmidtea mediterranea*. Planarians are bilaterally symmetric metazoan flatworms that contain a population of pluripotent, proliferative adult stem cells called neoblasts. Found throughout the body, the neoblasts function to replace cells lost during normal physiological turnover and upon injury making these organisms an excellent model for studying genes involved in the early stages of converting a normal stem cell to a cancerous cell [6], [7].

For the purpose of this study, we focused on *Smed-msh2* since its human homolog is commonly mutated in LS [8] and because of its involvement in damage recognition [9]. We exposed animals depleted of *Smed-msh2* by RNA-interference (RNAi) to a DNA alkylating agent, a potent inducer of MMR-dependent cell death *in vitro* [10], and determined that planarians lacking MMR were more tolerant to DNA damage than control animals and thus displayed increased survival. Similarly, we observed improved animal regeneration and an increased presence of mitotic cells after DNA damage in *Smed-msh2* deficient animals, suggesting that loss of MMR provides adult stem cells with an *in vivo* survival advantage in this cytotoxic environment.

## Results

### Identification of planarian MMR homologs

**Figure 1:**
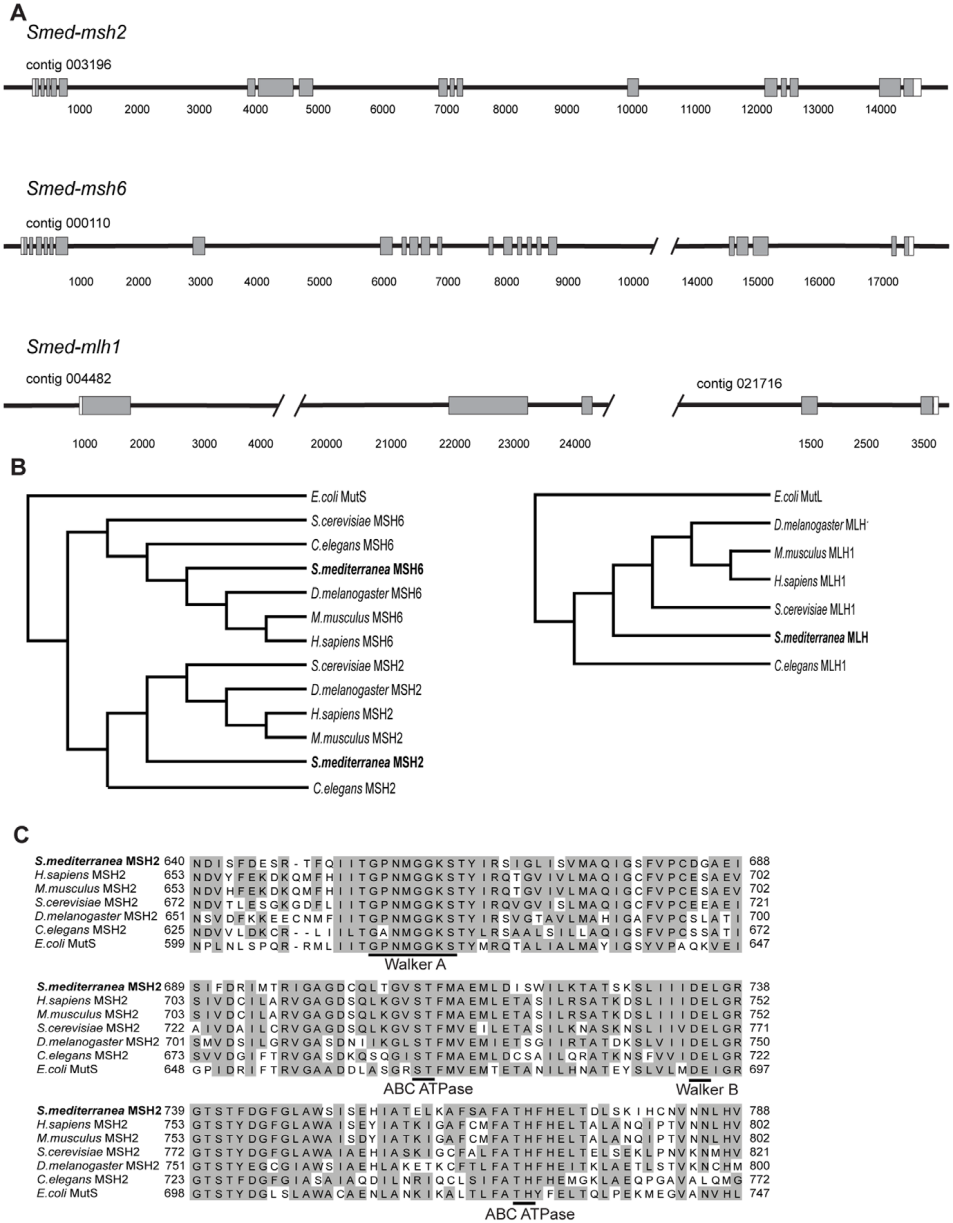
The planarian mismatch repair homologs. (A) Exon/intron map of the *Smed-msh2, Smed-msh6,* and *Smed-mlh1* genes. The exons are depicted by rectangles (ORF = shaded; UTRs = unshaded). Contig numbers are from the *S. mediterranea* Genome Database (18). *Smed-mlh1* spans two non-overlapping contigs. (B) Phylogenetic trees depicting the evolutionary relationship among the full-length MMR proteins encoded in genomes of common model organisms using the Neighbor joining method. (C) Alignment of the ATPase domain of several MSH2 family members, with homologous residues shaded in gray.

The DNA MMR proteins have been extensively studied across many evolutionarily divergent model organisms. In order to find MMR family members in planarians, the human MMR protein sequences MSH2, MSH6, and MLH1 were used to search the draft assembly of the *S. mediterranea* genome [11]. TBLASTN [12] revealed significant similarities from which we were able to design gene-specific primers and clone fragments of each gene. 5′ and 3′ RACE were used to clone full-length cDNA sequences for *Smed-msh2* (GenBank accession number JF511467), *Smed-msh6* (GenBank accession number JF519637), and *Smed-mlh1* (GenBank accession number JF511468), including their respective 5′ and 3′ UTRs (Figure 1A). The three MMR genes cluster with other MSH and MLH homologs phylogenetically (Figure 1B and S1). Gene ontology annotations [13] predict that *Smed-msh2*, *Smed-msh6* and *Smed-mlh1* contain domains associated with ATP binding, mismatched DNA binding and MMR. The predicted planarian Msh2 protein reveals perfect identity at all four adenosine nucleotide-binding motifs conserved in the MutS family that are necessary for proper function (Figure 1C).

**Figure 2:**
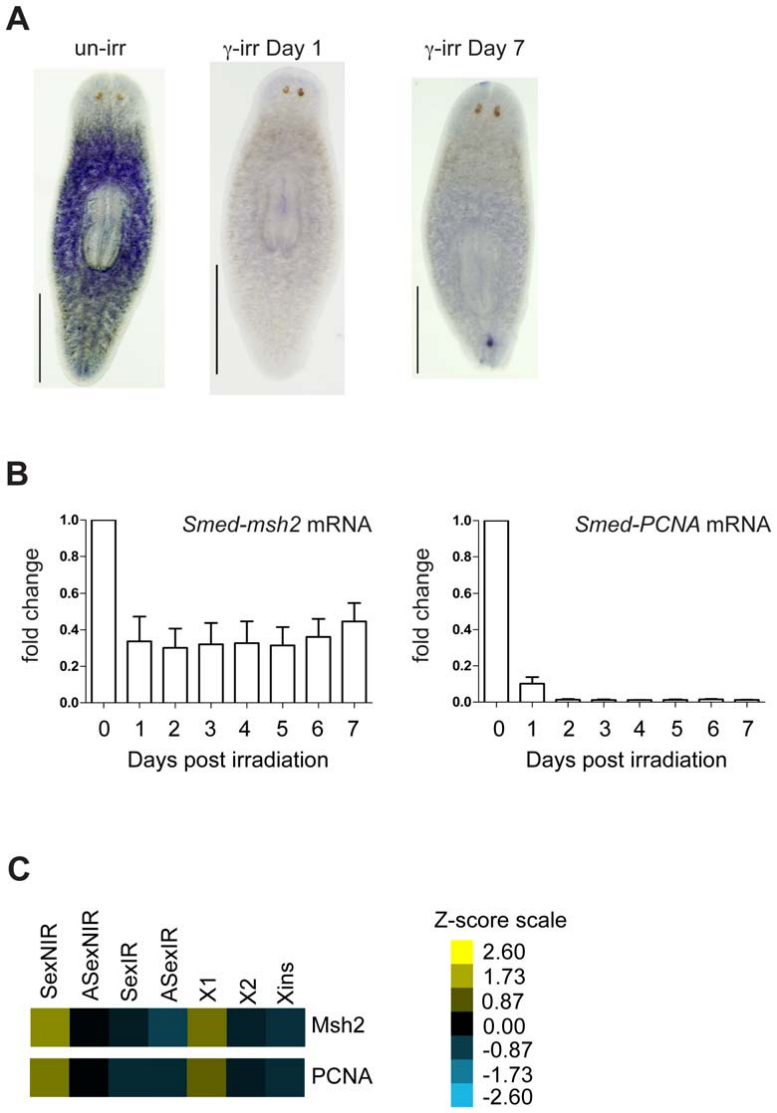
*Smed-msh2* expression in neoblasts. (A) Whole-mount in situ hybridization depicting the expression pattern of *Smed-msh2* in planarians. Anterior end at top. Scale bars: 0.5 mm. (B) The expression levels of *Smed-msh2* and *Smed-PCNA* mRNA examined by qRT-PCR using RNA isolated from five unirradiated or γ-irradiated asexual animals. Error bars indicate SD from three experiments. The y-axis fold change is normalized to *Smed-GAPDH*. (C) Transcript expression profiles of non-irradiated (NIR) and irradiated (IR) sexual or asexual strains (columns 1-4) and adult cell populations (columns 5-7, see text for definitions). Raw expression levels (FPKM) were standardized and converted into Z-scores which measure the number of standard deviations of each value above or below the mean. The color range for Z-scores is indicated in yellow and blue, respectively.

The distribution of *Smed-msh2* expression as determined by whole-mount in situ hybridization (WISH) is similar to other known neoblast markers including *smedwi-2* [14] and *Smed-PCNA* [15]. The expression is primarily posterior to the photoreceptors and excluded from the highly differentiated pharynx (Figure 2A). We exposed planarians to gamma irradiation to specifically eliminate neoblasts and their progeny, leaving post-mitotic tissues unaffected [16], [17]. Following irradiation, a dramatic decrease in *Smed-msh2* expressing cells was detected within 24 hours, consistent with expression localized to the neoblasts (Figure 2A). Weak uniform staining remained after irradiation suggesting *Smed-msh2* is expressed in some non-neoblasts. We confirmed this by examining *Smed-msh2* mRNA levels from irradiated animals using qRT-PCR. We detected a ∼2.5-fold decrease in *Smed-msh2* expression one day following irradiation that remained constant for at least 7 days post irradiation (Figure 2B). Though the timing of *Smed-msh2* expression loss was similar to *Smed-PCNA*, the extent of the loss was not as dramatic. Our findings were further validated using RNA-Seq data generated from RNA isolated from non-irradiated (NIR) and irradiated (IR) sexual and asexual animals. We found that expression levels of *Smed-msh2* and *Smed-PCNA* mRNA decreased in both strains following irradiation (Figure 2C). Moreover, RNA-Seq data from FACS purified irradiation-sensitive proliferating neoblasts (X1), irradiation-sensitive non-proliferating neoblasts (X2) and irradiation insensitive differentiated cells (Xins) revealed that *Smed-msh2* and *Smed-PCNA* are enriched in proliferating neoblasts (Figure 2C). Interestingly, we observed a reduced expression of *Smed-Msh2* and *Smed-PCNA* in asexual animals compared to sexual animals. Taken together, these results suggest that *Smed-msh2* is predominantly expressed in the stem cell population, though it is also expressed in some non-neoblast cell types as well.

### Smed-msh2 RNAi

**Figure 3:**
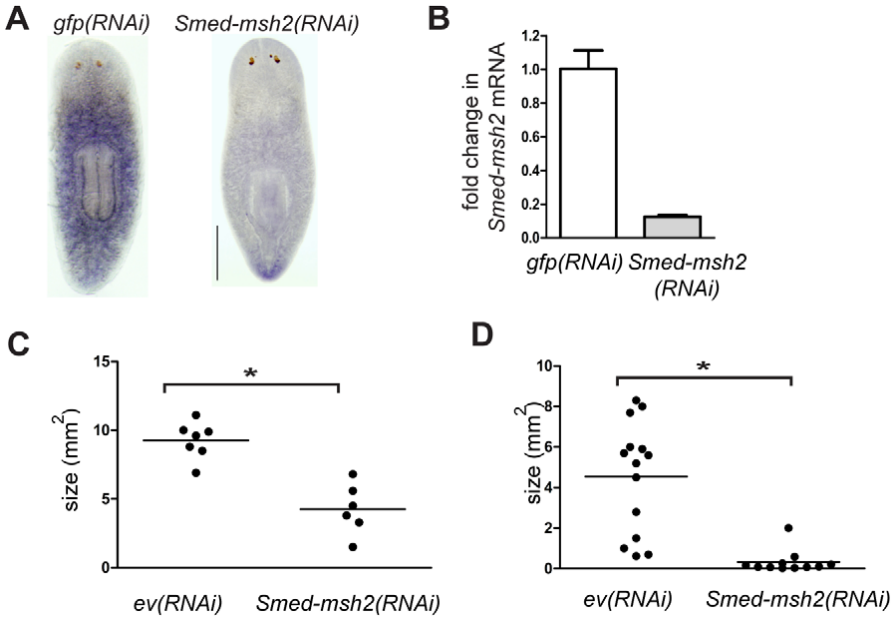
Long-term depletion of *Smed-msh2* by RNAi leads to shrinkage of animals. (A) WISH of *Smed-msh2* in *gfp(RNAi)* or *Smed-msh2(RNAi)* planarians. Dorsal view with anterior end at top. Scale bar: 0.5 mm. (B) qRT-PCR of *gfp(RNAi)* and *Smed-msh2(RNAi)* animals. Expression analyzed seven days after the final RNAi feeding. The y-axis fold change normalized to *smed-GAPDH*. Error bars indicate SD from samples in triplicate. (C). Four planarians per group were initially fed *Smed-msh2(RNAi)* or *ev(RNAi)* as described under *Methods*. Animals were fed at weekly intervals beginning at one month following first feeding. Animals were bisected at seven weeks and again at ten weeks. After four months, animals were photographed and measured using ImageJ software. (D). Ten planarians per group were initially fed *Smed-msh2(RNAi)* or *ev(RNAi)* as described under *Methods* then injected with DMSO on day six after the first feeding. Animals were fed at weekly intervals beginning at five weeks following first feeding. One *ev(RNAi)* and three *Smed-msh2(RNAi)* animals died for reasons unrelated to treatment. Additional animals were randomly removed for verification of RNAi knockdown. After three months, remaining animals were photographed and measured using ImageJ software. Statistical differences are measured by Student's *t-*test (*indicates p<0.05).

Adult planarians that are fed bacterially-expressing dsRNA targeted to a gene of interest results in specific mRNA knockdown that efficiently spreads to all tissues throughout the organism [18]. Following RNAi feedings, WISH and qRT-PCR confirmed that *Smed-msh2* was efficiently reduced (Figures 3A and 3B). *Smed-msh2(RNAi)* planarians were monitored for one month following treatment and did not exhibit any apparent phenotypes that differed from empty-vector fed RNAi [*ev(RNAi)*] or *gfp(RNAi)* controls suggesting that *Smed-msh2* is not required for normal tissue homeostasis. To examine whether loss of *Smed-msh2* had an effect on neoblasts, we bisected the *Smed-msh2(RNAi)* animals and monitored regeneration. Upon amputation, planarians are able to completely regenerate within 17 days in a process that is dependent on functioning adult stem cells [7], [19]. We observed that *Smed-msh2(RNAi)* animals regenerated completely similar to controls. These results were not surprising as constitutive loss of Msh2 in mice results in live animals that appear to develop normally [20]. However, these mice succumb to cancer at around six months of age. To determine the long-term effects of loss of *Smed-msh2* on planarians, we continued weekly *Smed-msh2(RNAi)* or *ev(RNAi)* feedings on small groups of animals and monitored the animals for an additional three months. At approximately seven and ten weeks following initial RNAi feeding, we bisected the animals to determine any regeneration defects. All amputated fragments from *Smed-msh2(RNAi)* and *ev(RNAi)* groups regenerated completely within two weeks at both time points. After the second round of regeneration, the control animals resumed normal growth, but the *Smed-msh2(RNAi)* animals appeared to shrink. By the end of the fourth month, two of the *Smed-msh2(RNAi)* animals died. The remaining *Smed-msh2(RNAi)* animals were significantly smaller than the *ev(RNAi)* control animals (Figure 3C). These remaining *Smed-msh2(RNAi)* animals died shortly after by lysis, similar to animals treated with gamma-irradiation. We observed a similar shrinking phenotype with a second group of *Smed-msh2(RNAi)* animals that were fed weekly for three months following injection with DMSO (as a negative control for experiments described below). These animals were never cut, however, the *Smed-msh2(RNAi)* animals shrunk considerably compared to the *ev(RNAi)* control animals (Figure 3D).

### Effect of DNA damage on *Smed-msh2(RNAi)* animals

**Figure 4:**
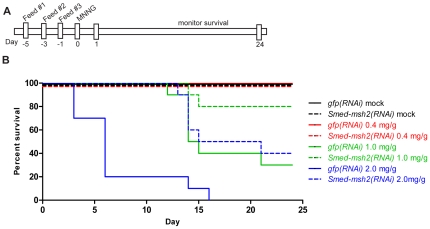
Depletion of *Smed-msh2* confers tolerance to DNA damage. (A) Experimental timeline for MNNG survival experiments. (B) Survival curves of *gfp(RNAi)* and *Smed-msh2(RNAi)* planarians in response to indicated doses of MNNG generated using GraphPad Prism 5. n = 10 worms per group. Differences between *gfp(RNAi)* and *Smed-msh2(RNAi)* curves are significant for 1.0 mg/g and 2.0 mg/g doses (P = 0.024 and 0.0007, respectively, using log-rank test).

MMR-deficient tumor cell lines and mouse embryonic fibroblasts (MEFs) from *Msh2, Mlh1* and *Msh6* knockout mice are less sensitive to treatment with the DNA alkylating agent N-methyl-N'-nitro-N-nitrosoguanidine (MNNG) [10], [21], [22], [23], [24], [25]. Based on this *in vitro* data from higher organisms, we predicted that loss of *Smed-msh2* would result in an increased survival advantage in planarians treated with MNNG. To test the effects of alkylation damage on planarians, animals were depleted of *Smed-msh2* as described above. 24 hours after final RNAi feeding, the animals were injected with varying doses of MNNG or a DMSO-only control and monitored for the next 24 days (Figure 4A). At the lowest dose of MNNG (0.4 mg/g) 100% of control and *Smed-msh2(RNAi)* animals survived the duration of the experiment. However, after exposure to 1.0 mg/g MNNG, a significant survival advantage was observed for the *Smed-msh2(RNAi)* animals compared to the *gfp(RNAi)* animals (P = 0.024, log rank test) (Figure 4B). Similarly, at the highest dose of MNNG (2.0 mg/g), 100% of the *gfp(RNAi)* animals died by day 16 while the *Smed-msh2(RNAi)* animals showed an overall increase in survival with a marked delay in death among the animals that did succumb to the drug (P = 0.0007, log rank test).

MNNG treatment results in the addition of methyl groups to multiple nucleophilic sites on DNA bases including the O^6^ position of guanine. If left unrepaired, the O^6^-MeG will mismatch with thymine during DNA replication [26]. The cytotoxicity of O^6^-MeG:T mismatches depends upon their recognition by MSH2-MSH6. To confirm that our *Smed-msh2(RNAi)* animals are more resistant to MNNG due to a MMR-dependent recognition of O^6^-MeG lesions, we treated these animals with another DNA alkylating agent, methylmethane sulfonate (MMS). MMS generates a significantly smaller percentage of O^6^-MeG lesions than MNNG, thus its cytotoxicity does not depend upon recognition by MSH2-MSH6 [26]. *Smed-msh2(RNAi)* and *gfp(RNAi)* animals were treated with increasing doses of MMS and monitored for ten days after treatment. In contrast with MNNG, there was no survival advantage observed for *Smed-msh2(RNAi)* animals compared to *gfp(RNAi)* controls (Figure S2). There was a modest survival disadvantage for *Smed2-msh2(RNAi)* animals at the intermediate dose (500 µM) of MMS that was significant (P = 0.015, log rank test). These results combined with the MNNG survival data are consistent with a mechanism in which the Smed-msh2 protein is involved in an O^6^-MeG-dependent cell death process.

**Figure 5:**
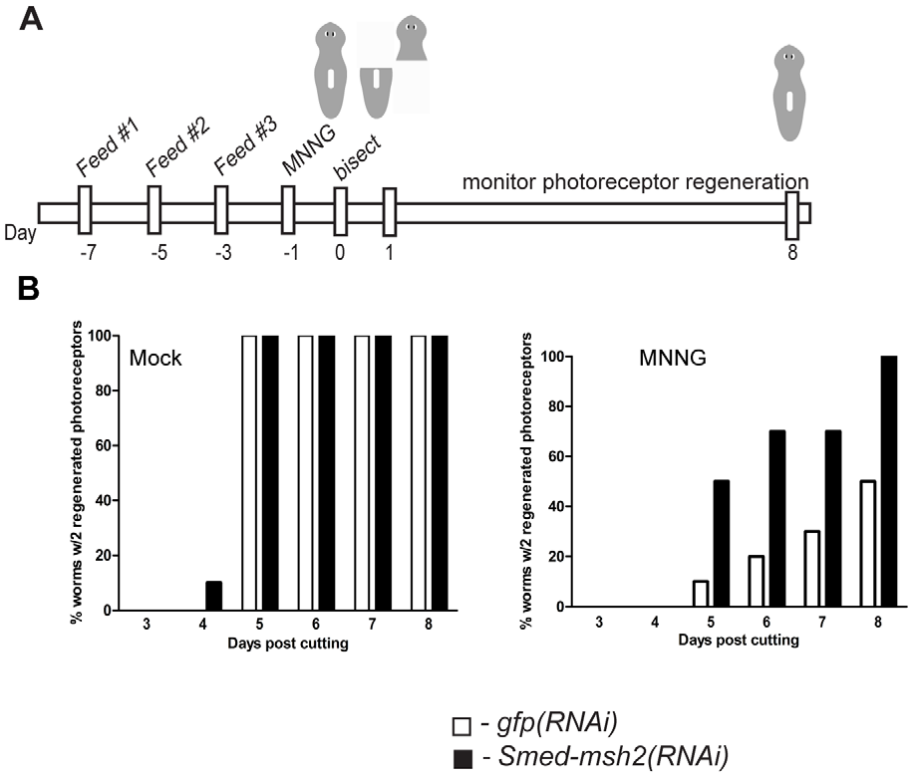
*Smed-msh2*-dependent regeneration defect in MNNG treated planarians. (A) Experimental timeline for analyzing regeneration of *gfp(RNAi)* and *Smed-msh2(RNAi)* planarians following MNNG treatment. (B) Percentage of animals with complete regeneration of two photoreceptors on dorsal fragment following bisection of mock (left panel) or 1.0 mg/g MNNG (right panel) treated animals. White bars: *gfp(RNAi).* Black bars: *Smed-msh2(RNAi)*. (n = 10 worms/group)

In order to determine whether the observed survival advantage following MNNG treatment was the result of a stem-cell specific response, we took advantage of the regenerative capabilities of the planarian. To measure stem cell function, we examined the effects of alkylation damage on regeneration in RNAi treated animals. Following RNAi feedings as described above, planarians were injected with 1.0 mg/g MNNG and bisected laterally 24 hours later (Figure 5A). As a marker for regeneration, we monitored photoreceptor developments in regenerating tail blastemas. Regeneration of the photoreceptors occurred by day 5 in 100% of mock injected *gfp(RNAi)* and *Smed-msh2(RNAi)* animals (Figure 5B). We observed a dramatic inhibition of regeneration in *gfp(RNAi)* animals injected with MNNG. Only one animal had regenerated photoreceptors by day 5, with half of the animals failing to regenerate at all. Though slightly delayed, all of the *Smed-msh2(RNAi)* animals injected with MNNG regenerated photoreceptors. These results suggest that loss of *Smed-msh2* provides the stem cells a survival advantage in the presence of DNA damage.

**Figure 6:**
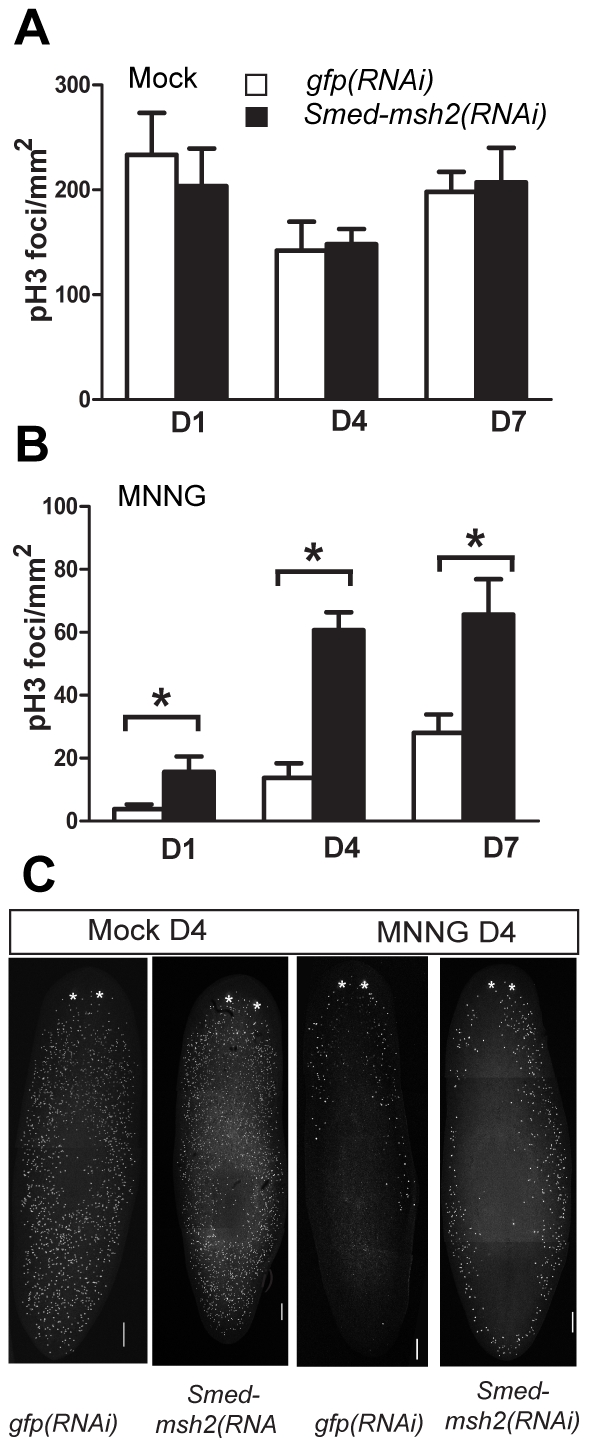
*Smed-msh2* depletion enhances survival of mitotically active neoblasts in MNNG treated planarians. The number of H3ser10 p (pH 3) stained foci per mm2 as a marker of mitotically active neoblasts in mock (A) and MNNG (B) treated planarians. *gfp(RNAi)* and *Smed-msh2(RNAi)* animals were stained following exposure to 1.0 mg/g MNNG at days 1, 4, and 7-post injection. White bars: *gfp(RNAi).* Black bars: *Smed-msh2(RNAi)*. Statistical differences measured by Student's *t-*test and error bars indicate SEM. (* indicates p<0.05) n = at least four animals per experiment with two experimental replicates. (C) Representative animals from day four time point. Dorsal view with anterior end at top. * indicates location of photoreceptors. Scale bars: 200 µm.

To further confirm that MNNG resulted in the elimination of the dividing stem cells, we utilized the presence of phosphorylated histone H3 (pH 3) as a marker for mitotically active cells. Histone H3 is phosphorylated on its amino terminus as cells traverse from the G_2_ to M phase of the cell cycle [27]. Both *gfp(RNAi)* and *Smed-msh2(RNAi)* animals were exposed to MNNG and then fixed 1, 4 or 7 days later. We observed a dramatic decrease in the number of pH 3-positive cells following MNNG treatment in both groups of animals. However, we consistently noticed a significantly greater number of pH 3 stained cells in the *Smed-msh2(RNAi)* animals at each time point (p<0.05) (Figures 6A and 6B). These results suggest that MMR-defective cells are less sensitive to growth arrest and cell death effects of alkylation damage *in vivo*.

## Discussion

When the MMR genes were first linked to Lynch syndrome, it was proposed that loss of MMR results in a cell with a mutator phenotype that accumulates mutations in important oncogenes and tumor suppressors at an increased rate [2]. However, the ability of the MMR proteins to invoke cell cycle checkpoints and cell death in response to certain DNA lesions indicate that this pathway may be involved in a more global DNA damage response that prevents the propagation of cells with damaged DNA. This global damage response has been proposed to serve as a barrier against genetic instability that must be overcome for the cell to progress to malignancy [28], [29].

Though much has been learned about MMR function in cell culture models, to understand the role of MMR in tumorigenesis, *in vivo* studies are necessary. Animal models of MMR deficiency including mouse models have been very useful in this regard [20]. In particular, two mouse models that carry missense mutations in *Msh2* or *Msh6* have provided some interesting clues about the role of the MMR-dependent damage response in tumorigenesis [30], [31]. Mouse embryonic fibroblasts carrying these mutations were shown to be inefficient at repairing DNA mismatches, yet maintain the MMR-dependent sensitivity to certain cytotoxic agents. Interestingly, though these mice develop cancer, the onset of tumor formation appears significantly delayed compared to complete gene knockout mice. These results suggest that, though the establishment of a mutator phenotype is likely the main driving force for tumorigenesis in MMR-defective cells, the ability of the MMR proteins to maintain a global DNA damage response may also play a role in delaying tumorigenesis.

We have previously hypothesized that loss of MMR function in an adult stem cell may provide a temporary competitive advantage allowing for the increased propagation of daughter cells that carry a mutator phenotype [32]. To address this hypothesis, we wanted to examine the response of MMR-deficient cells to stress prior to tumor formation. We utilized the planarian *S. mediterranea* as an *in vivo* animal model due to the relative ease with which one can monitor a population of adult stem cells in this organism. *Smed-msh2(RNAi)* animals display increased tolerance to the cytotoxic effects of the DNA damaging drug MNNG compared to control animals. In addition, we observed a delayed, but otherwise normal, regeneration response in *Smed-msh2(RNAi)* animals exposed to MNNG compared to *gfp(RNAi)* controls. Our pH 3 foci studies also revealed that MMR-defective neoblasts are significantly more resistant to the effects of DNA damage than normal stem cells. We did observe an overall decrease in pH 3-positive cells in both the *Smed-msh2(RNAi) and gfp(RNAi)* animals after MNNG treatment. This result likely reflects the ability of MNNG to activate MMR-independent checkpoint and cell death pathways, possibly through activation of the base excision repair pathway [33]. An increase in mitotic activity over time is observed in both populations of animals suggesting not all of the neoblasts are destroyed by the damage, however, the increase is significantly greater in the *Smed-msh2(RNAi)* group at every time point. It is possible that the differences we observe would be more dramatic if loss of MMR expression were complete such as through deactivating mutations. The ability to generate transgenic planarians would greatly enhance these studies.

A prediction from our results is that the surviving stem cells in the *Smed-msh2(RNAi)* animals would continue to accumulate mutations perhaps leading to tumor formation. There is evidence to suggest that planarians can develop tumors when treated with carcinogens [34]. We examined a population of *Smed-msh2(RNAi)* animals for four months for signs of tumor formation. We did not observe any noticeable signs of tumor growth, however, interestingly the *Smed-msh2(RNAi)* animals became increasingly smaller compared to *ev(RNAi)* control animals. These results may be a phenomenon limited to the planarian. Planarians are capable of regulating neoblast division in response to its environment. When nutrients are scarce, for example, planarians will shrink due to a change in cell number [35]. This may result from an increase in apoptosis or possibly a loss of stem cell asymmetric division in which both daughters become post-mitotically differentiated cells. We are currently examining these possibilities in long-term *Smed-msh2(RNAi)* fed animals. This ability to regulate total cell number may provide an extra anti-tumorigenic protection in this organism. Alternatively, these results may suggest that while loss of MMR can provide a short-term selective advantage under certain environmental conditions, loss of MMR in the long term is disadvantageous.

## Methods

### Planarian culturing

Asexual and sexual strains of *S. mediterranea* were maintained essentially as described [36] at 20°C in ddH_2_O supplemented with 1.6 mM NaCl, 1.0 mM CaCl_2_, 1.0 mM MgSO4_4_, 0.1 mM MgCl_2_, 0.1 mM KCl, and 1.2 mM NaHCO_3_ and fed homogenized calf liver. All animals were starved for one week prior to any experiments.

### Cloning mismatch repair genes

Putative MMR genes were identified by performing TBLASTN searches of the version 3.1 draft assembly of the *S. mediterranea* genome with human MMR proteins. The *Smed-msh2, Smed-mlh1,* and *Smed-msh6* cDNAs were obtained by RT-PCR cloning of an internal fragment, followed by 5′ and 3′ Rapid Amplification of cDNA Ends using the FirstChoice RLM-RACE kit (Ambion, Inc.).

### Gene Expression Analysis

Expression profiles for MMR genes were analyzed using RNA-Seq data to be described elsewhere (A.M.R., D. P. and B.R.G., in preparation). Briefly, reads were aligned to the draft assembly of the *S. mediterranea* genome using Bowtie alignment software [37]. Read densities were used to measure transcript expression levels in units of FPKM (fragments per kilobase of exon model per million mapped reads) [38]. X1, X2 and Xins cell populations were isolated from sexual planarians by fluorescent activated cell sorting as previously described [39]. Transcript expression levels between irradiated and non-irradiated samples and X1, X2 and Xins cell populations were compared using Z-scores.

### RNAi

cDNAs for individual genes were cloned into pDONRdT7 [40] using a BP reaction (Invitrogen) and the resulting clones transformed into *Escherichia coli* strain HT115. 2.0 mL of bacterial culture was pelleted and resuspended in 25 µL of 1∶1 homogenized calf liver and water, and mixed with 0.7 µL of red food coloring to form a paste. Animals were fed RNAi every other day for a total of three feedings. For long-term RNAi experiments, animals were initially fed as described above, then fed once per week for the duration of the experiment.

### Quantitative Reverse Transcriptase PCR

First strand cDNA was synthesized from 2 µg of total planarian RNA using SuperScriptII (Invitrogen). Total RNA was DNase I (Amp Grade, Invitrogen) treated prior to reverse transcription. Gene specific primers were designed for the individual candidate genes using the Custom TaqMan® Assay Design Tool (Applied Biosystems). All qRT-PCR experiments were performed in triplicate using an Applied Biosystems StepOne™ Real-Time PCR system. For RNAi experiments, the Standard Curve Method was used to determine the level of gene expression, using the ubiquitously expressed *Smed-GAPDH* (H.8.10b) as a reference. For irradiation experiments, planarians were exposed to 90 Gy of γ-irradiation using a Gamma Cell 1000 Cesium 137 irradiator at ∼200 rad/min for 45 min. The Comparative ΔΔC_t_ Method was used to determine gene expression levels of *Smed-msh2* and *Smed-PCNA*.

### Survival and Regeneration Assays

RNAi was performed on groups of ten sexual animals [41]. One day following the last RNAi feeding (as described above), animals were injected once with the indicated concentrations of MNNG (obtained from the National Cancer Institute Chemical Carcinogen Reference Standard Repository; CAS: 70-25-7) dissolved in DMSO mixed with red food coloring. For the survival assay, animals were monitored for viability for 24 days following injection. Alternatively, RNAi treated animals were soaked in the indicated concentrations of MMS (Sigma) for ten days and monitored daily for viability. For regeneration assays, animals were bisected laterally 24 hours after 1.0 mg/g MNNG or vehicle control injection and tail fragments were monitored for appearance of both photoreceptors.

### Immunohistochemistry and whole-mount in situ hybridization (WISH)

Immunostaining with anti-phosphohistone H3 (pH 3) antibody (Cell Signaling) was performed as described [40]. Specimens were imaged on a Zeiss LSM 510Meta confocal microscope. Images were processed and quantified using ImageJ (http://rsbweb.nih.gov/ij/). WISH was performed essentially as described [42]. Hybridization was carried out at 55°C in prehybridization buffer supplemented with 2% dextran sulfate. Animals were developed using BM Purple (Roche).

## Supporting Information

Figure S1 ***Smed-Msh2*** **clusters with other** ***MSH2*** **homologs.** Phylogenetic tree depicting the evolutionary relationship among the full-length MutS protein homologs encoded in genomes of common model organisms using the Neighbor joining method.
(TIF)

Figure S2 **Depletion of** ***Smed-msh2*** **confers no selective advantage to MMS.** Survival curves of *gfp(RNAi)* and *Smed-msh2(RNAi)* planarians in response to indicated doses of MMS generated using GraphPad Prism 5. n = 10 worms per group. Differences between *gfp(RNAi)* and *Smed-msh2(RNAi)* curves are significant for 500 µM MMS curves (P = 0.015, using log-rank test).
(TIF)
