# Supplementary figures and images for: Loss of DNA Mismatch Repair Imparts a Selective Advantage in Planarian Adult Stem Cells
Source: PLoS One. 2011 Jul 1;6(7):e21808. doi: 10.1371/journal.pone.0021808 (PMC3128615; doi:10.1371/journal.pone.0021808)

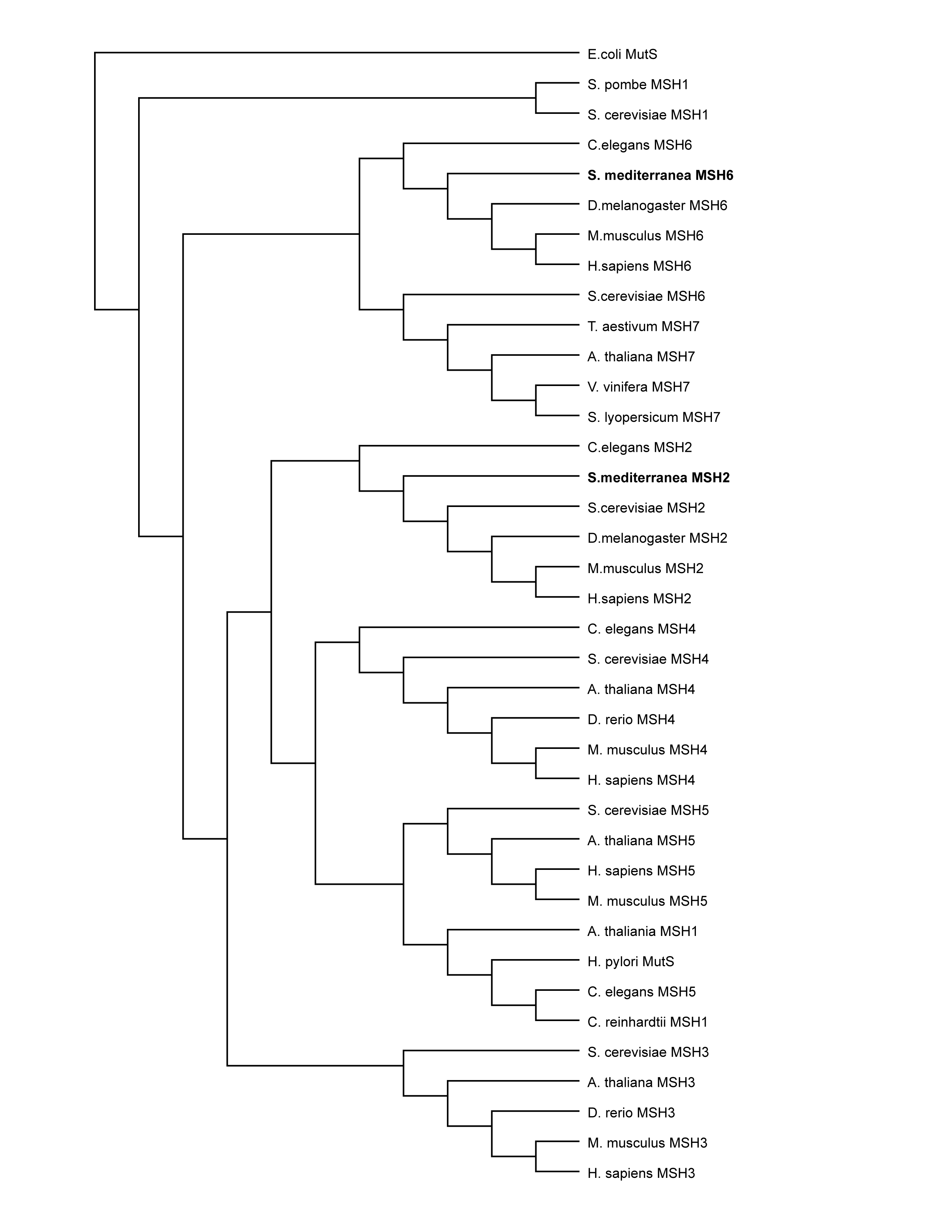

Supplement: Figure S1 — Smed-Msh2 clusters with other MSH2 homologs. Phylogenetic tree depicting the evolutionary relationship among the full-length MutS protein homologs encoded in genomes of common model organisms using the Neighbor joining method. (TIF) [file pone.0021808.s001.tif]

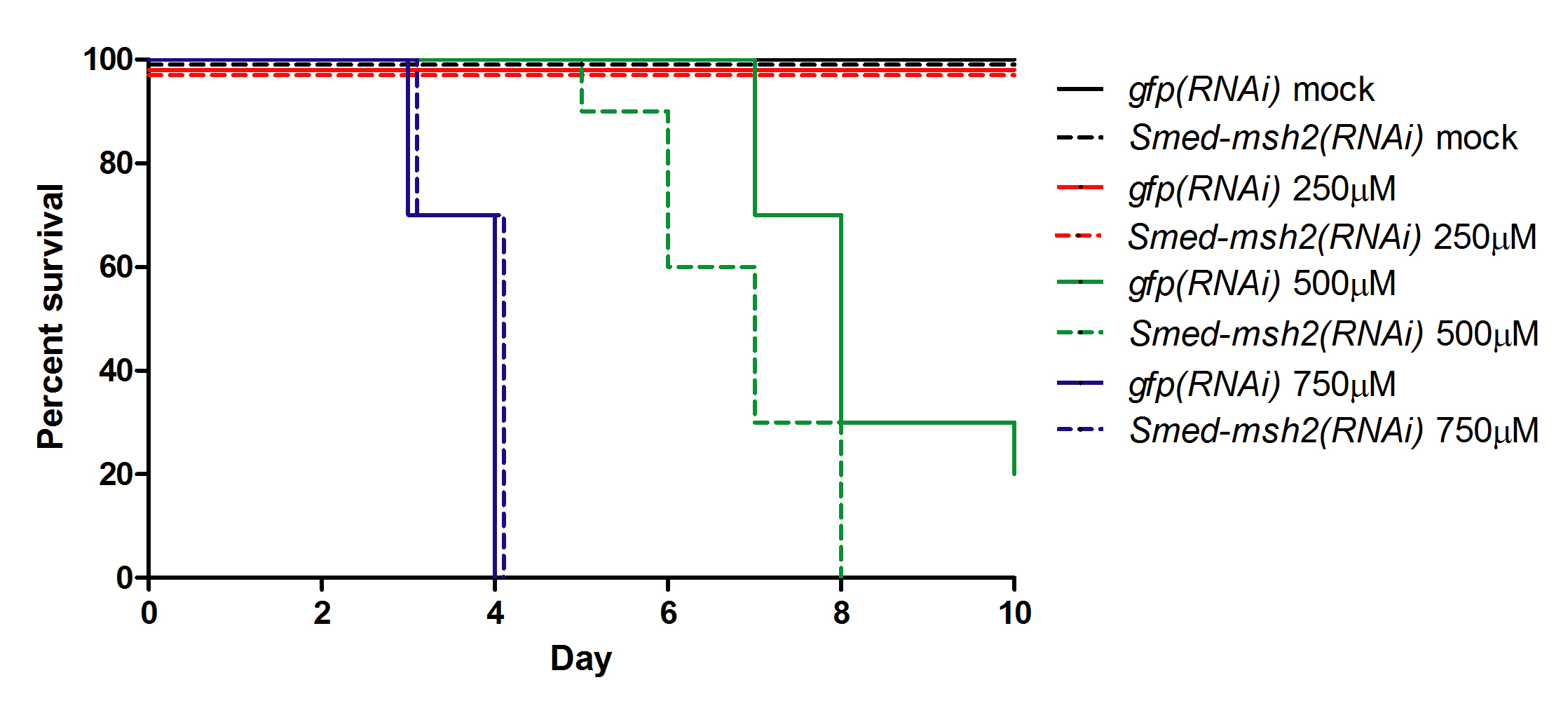

Supplement: Figure S2 — Depletion of Smed-msh2 confers no selective advantage to MMS. Survival curves of gfp(RNAi) and Smed-msh2(RNAi) planarians in response to indicated doses of MMS generated using GraphPad Prism 5. n = 10 worms per group. Differences between gfp(RNAi) and Smed-msh2(RNAi) curves are significant for 500 µM MMS curves (P = 0.015, using log-rank test). (TIF) [file pone.0021808.s002.tif]
